# Development of Biomarker Signatures Associated with Anoikis to Predict Prognosis in Endometrial Carcinoma Patients

**DOI:** 10.1155/2021/3375297

**Published:** 2021-12-28

**Authors:** Shan Chen, Jiamin Gu, Qinfen Zhang, Yan Hu, Yu Ge

**Affiliations:** Department of Obstetrics and Gynecology, Zhongda Hospital, Southeast University, Nanjing, China

## Abstract

**Purpose:**

To generate a signature based on anoikis-related genes (ARGs) for endometrial carcinoma (EC) patients and elucidate the molecular mechanisms in EC.

**Methods:**

On the basis of TCGA-UCEC dataset, we identified specific anoikis-related genes (ARGs) in EC. Cox-relative regression methods were used to generate an anoikis-related signature (ARS). The possible biological pathways of ARS-related genes were analyzed by GSEA. The clinical potency and immune status of ARS were analyzed by CIBERSORT method, ssGSEA algorithm, Tumor Immune Dysfunction and Exclusion (TIDE) analysis. Moreover, the expression patterns of ARS genes were verified by HPA database.

**Results:**

Seven anoikis genes (CDKN2A, E2F1, ENDOG, EZH2, HMGA1, PLK1, and SLC2A1) were determined to develop a prognostic ARS. Both genes of ARS were closely bound up with the prognosis of EC patients. The ARS could accurately classify EC cases with different clinical outcome and mirror the specific immune status of EC. We observed that ARS-high patients could not benefit from immunotherapy. Finally, all the hub genes of ARS were proved to be upregulated in EC tissues by immunohistology.

**Conclusion:**

ARS can be used to stratify the risk and forecast the survival outcome of EC patients and provide prominent reference for individualized treatment in EC.

## 1. Introduction

Endometrial carcinoma is one of the most common malignant tumors in the female reproductive system [1]. Its incidence rate is increasing year by year, showing a younger trend. Studies have shown that, about 76,000 women worldwide die of endometrial cancer each year, and its high mortality and morbidity have become an important factor threatening women's health [2]. Although, EC patients at early stage display a favorable clinical outcome, more than 30% of cases have distant metastasis with a lower survival rate [3]. Given the shortcomings of a single gene for the assessment of prognosis, uncovering the underlying mechanism of metastasis and determining reliable multibiomarkers to forecast the prognosis of EC patients are extremely urgent.

In recent years, several studies reported that cancer cells carry extracellular matrix (ECM) during metastasis, and anoikis occurs when tumor cells detach from the ECM during metastasis. Anoikis is a specific form of cell apoptosis caused by the detachment of cells from the ECM. Originally identified in epithelial and endothelial cells, anoikis is thought to be a physiological process associated with development and tissue homeostasis. Apoptosis prevents detached cells from readhering to other substrates for abnormal proliferation and thus plays a central part in protecting the organism. However, the inability to initiate the loss-of-nest apoptosis program may result in the survival of adherent cells in suspension or proliferation in an ECM different from the in situ one. Currently, diminished ability to initiate apoptosis has become a hallmark of cancer and contributes to the development of distal metastases from tumor.

Several reports have verified that anoikis-related genes (ARGs) play a central part in tumor metastatic cascade and cancer progression, including gastric carcinoma (GC) [4], lung cancer (LC) [5], breast carcinoma (BC) [6], and EC [7]. She and his colleagues revealed that overexpression of FAIM2 is associated with dismal clinical outcome for lung cancer, and silencing FAIM2 may inhibit tumor cell viability and anoikis resistance [8]. KLF5, a novel prognostic biomarker in colorectal cancer, has been proved to regulate cell proliferation and anoikis resistance. In EC, L1CAM could facilitate epithelial mesenchymal transformation (EMT) by boosting the development of cancer initiating cells in HEC-1A cells, thereby promoting loss-of-nest apoptosis-resistant and affecting patient prognosis [7].

Although anoikis has been proved to be associated with prognosis in several tumors [5, 9, 10], prognostic indicators based on ARGs have been rarely analyzed in EC. Therefore, we focus on the relationship between integrated ARGs and EC clinical outcome. In our study, we identified a powerful ARG-based signature and exploit its clinical implications in EC patients.

## 2. Methods

### 2.1. Data Processing

By integrating the data of the TCGA-UCEC in The Cancer Genome Atlas (TCGA, https://portal.gdc.cancer.gov/), we obtained gene expression profiles and clinical data of 520 EC patients. A total of 434 anoikis-related genes (ARGs) were extracted from GeneCards, and genes with a relevance score >0.4 were selected (Supplementary Table S1). In order to obtain differentially expressed gene (DEG) in EC, we conducted differentiation analysis for the expression of all genes in the normal and tumor samples by limma in R software (|fold change (FC)| = 1.0 and *p* value <0.05). Then, differentially expressed ARGs were collected by interacting with DEG sets.

### 2.2. Identification of Anoikis-Related Signature

We randomly divided 520 EC patients into a training set and a test set at a ratio of 1 : 1 to develop an anoikis-related signature (ARS). Firstly, we obtained candidate prognostic genes in the training set through the univariate regression method based on the differentially expressed ARGs. Then, LASSO penalty analysis was employed to shrink the model of overfitting. Finally, we conducted multivariate Cox regression to set up a novel ARS. The risk score for each case was calculated using the following formula: risk factor = (expression of the ARG1 × coefficient) + (expression of the ARG2 × coefficient) + … + (expression of the ARGn × coefficient). According to the median value of the risk score, all cases were split into the high- and low-risk score group. In addition, the expression patterns of ARS model genes at protein levels were analyzed by Human Protein Atlas (HPA) website ((https://www.proteinatlas.org/) [11].

### 2.3. Establishment of an ARS-Based Nomogram

An independent prognostic analysis was performed by combining data from 520 patients with clinical information and risk score and using the “survival” package. Moreover, ARS-based nomogram was created based on risk score and other clinicopathological traits to forecast the clinical outcome of cases. Calibration curves were plotted to estimate the reliability of ARS by comparing the predictive power of the line graphs with the observed survival outcome.

### 2.4. Gene Set Enrichment Analysis (GSEA)

GSEA was applied to examine the biological pathways and immune activity associated with ARS based on the Hallmark and C7 gene sets v7.4. Enriched gene sets with *p* value <0.05 were collected after 1000 substitutions.

### 2.5. Immunity Analysis of the Signature

Based on TCGA RNA sequencing data, we quantified 22 types of immune cell proportion using the CIBERSORT tool. *P* value <0.05 was set as the threshold. In addition, we performed Tumor Immune Dysfunction and Exclusion (TIDE, https://tide.dfci.harvard.edu/) method to assess the immunotherapy response of EC patients.

### 2.6. Statistical Analysis

R software (4.0.1) was used among all statistical analyses. Kaplan–Meier (K–M) analysis was applied to assess survival differences between the two groups. The reliability of the ARS models was tested using receiver operating characteristic (ROC) analyses. Statistical significance was set at *p* value <0.05 for all analyses.

## 3. Results

### 3.1. Determination of Differentially Expressed ARGs

**Figure 1:**
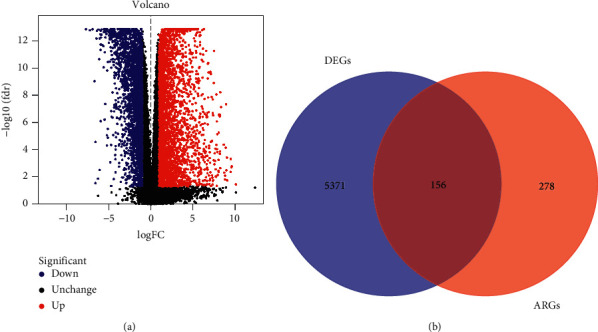
Characterization of differentially expressed anoikis-related genes (DEARGs). (a) Volcano plot of differentially expressed genes (DEGs) in endometrial carcinoma (EC). (b) Venn diagram of DEGs and ARGs.

The gene expression data of EC samples and normal control in TCGA were analyzed, and a total of 5527 DEGs were collected (Figure 1(a)). Next, these DEGs were overlapped with 434 ARGs extracted from GeneCards, resulting in 156 differentially expressed ARGs shared (Figure 1(b)).

### 3.2. Development and Validation of the ARS

**Figure 2:**
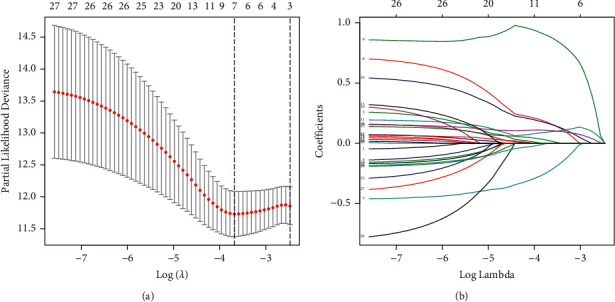
Identification of the anoikis-related signature (ARS). (a) LASSO regression analysis for the development of ARS. (b) LASSO coefficient of anoikis-related genes in EC.

We randomly divided 520 EC patients into a training set (*n* = 260) and a validation set (*n* = 260, Table 1). The univariate Cox method was employed to obtain 28 representative prognostic ARGs in the training set. Then, we conducted LASSO-penalized regression to eliminate the overfit gene of signature (Figures 2(a) and 2(b)). Moreover, a signature of seven ARGs (CDKN2A, E2F1, ENDOG, EZH2, HMGA1, PLK1, and SLC2A1) was set up through the multivariate cox analysis (Table 2). The constructed ARS was developed using the following formula: risk factor = (CDKN2A × (0.1068)) + (E2F1 × (0.0941)) + (ENDOG × (-0.2316)) + (EZH2 × (0.1626)) + (HMGA1 × (0.8872)) + (PLK1 × (0.1673)) + (SLC2A1 × (0.0228)]. Subsequently, a median value of risk score was utilized to spilt the cases into the high-risk score and low-risk score group.

**Figure 3:**
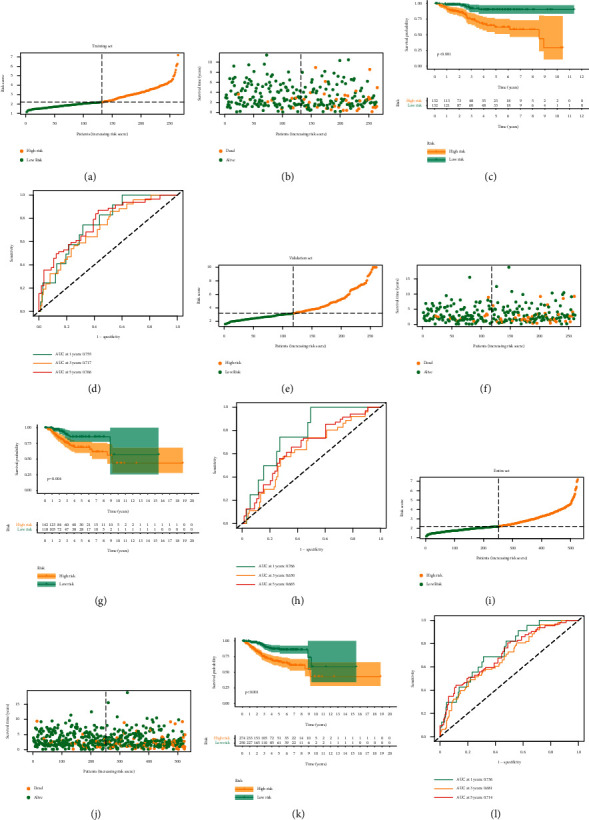
Predictive value of the ARS. (a) The layout of increasing risk scores in the training set. (b) The clinical outcome of EC cases in the training set. (c) Kaplan–Meier curves of survival outcome between two groups in the training set. (d) ROC curves of predictive performance of the ARS in the training set. (e–h) Similar results confirmed the validation and entire sets.

In the training set, our nominated ARS can discriminate risk score and clinical status of EC samples (Figures 3(a) and 3(b)). K–M analysis displayed that the ARS-high group had dismal clinical outcome (Figure 3(c)). The areas under the ROC curves (AUC) were 0.755, 0.717, and 0.766 for 1-, 3-, and 5-year survival, respectively (Figure 3(d)). The testing set and the entire cohort were applied to verify the predictive performance of the ARS. The layout of risk score and clinical status of EC patients in the testing and entire sets are shown in Figures 3(e) and 3(f) and Figures 3(i) and 3(j). The prognosis differences between the two risk groups were also confirmed in the testing and entire sets (Figures 3(g) and 3(k)). ROC methods indicated the predictive reliability of ARS in both testing and entire sets (Figures 3(h) and 3(l)).

### 3.3. Determination of a Nomogram

**Figure 4:**
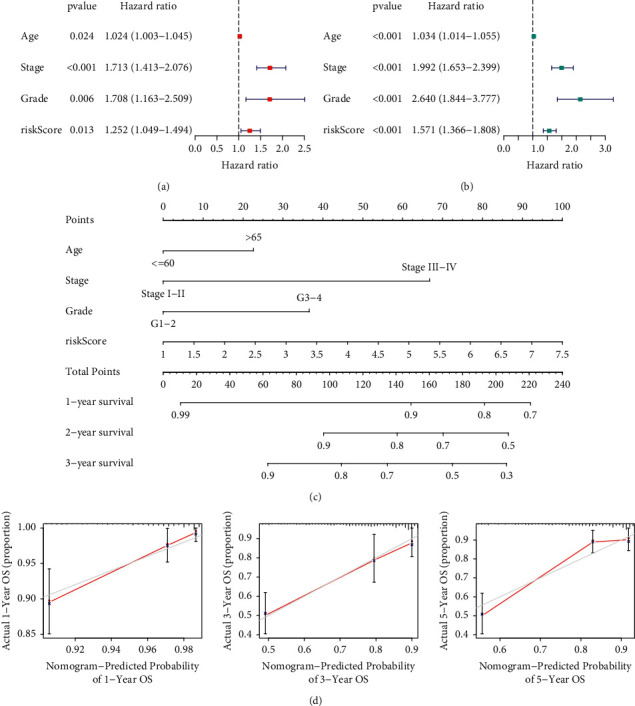
Construction of an ARS-based nomogram. Univariate (a) and multivariate cox regression (b) to estimate the independence of the ARS. (c) A nomogram generated based on the ARS. (d) Calibration curves showing favorable accuracy of the nomogram.

To determine the independence of the ARS model, the univariate and multivariate cox methods were applied. Univariate cox analysis disclosed that age, stage, grade, and risk score were meaningful prognostic indicators (Figure 4(a)). As revealed by the multivariate cox analysis, risk score was still meaningful for prognosis, indicating our proposed ARS can be independent of other clinical traits (Figure 4(b)). To further exploit the prognostic value of the ARS, a nomogram was established by combining the clinical factors and ARS model (Figure 4(c)). Afterward, the calibration curves of 1-, 3-, and 5-year survival time showed the nomogram had a powerful value for forecasting prognosis (Figure 4(d)).

### 3.4. GSEA Analysis of the ARS

**Figure 5:**
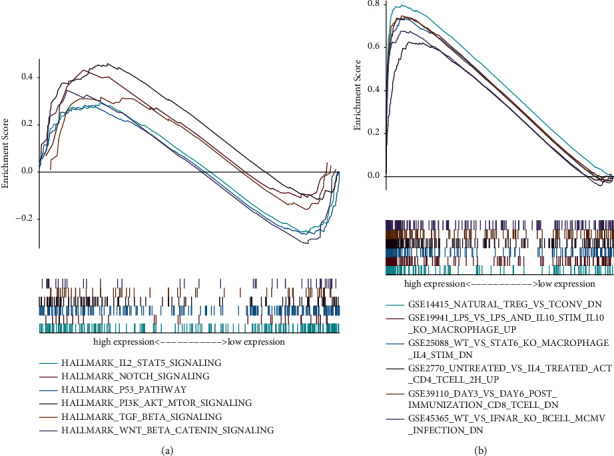
Gene set enrichment analysis. (a) Gene sets of hallmarks. (b) Gene sets of immune activity.

We found that the greatly enriched hallmarks were “IL2/STAT5 signaling,” “notch signaling,” “P53 signaling,” “PI3K/AKT/mTOR signaling,” “TGF *β* signaling,” and “Wnt/*β*-catenin signaling” (Figure 5(a)). In terms of C7 immune gene sets, various immune functions were enriched in the ARS-high group (Figure 5(b)).

### 3.5. Immune Activity Analysis of the ARS Model

**Figure 6:**
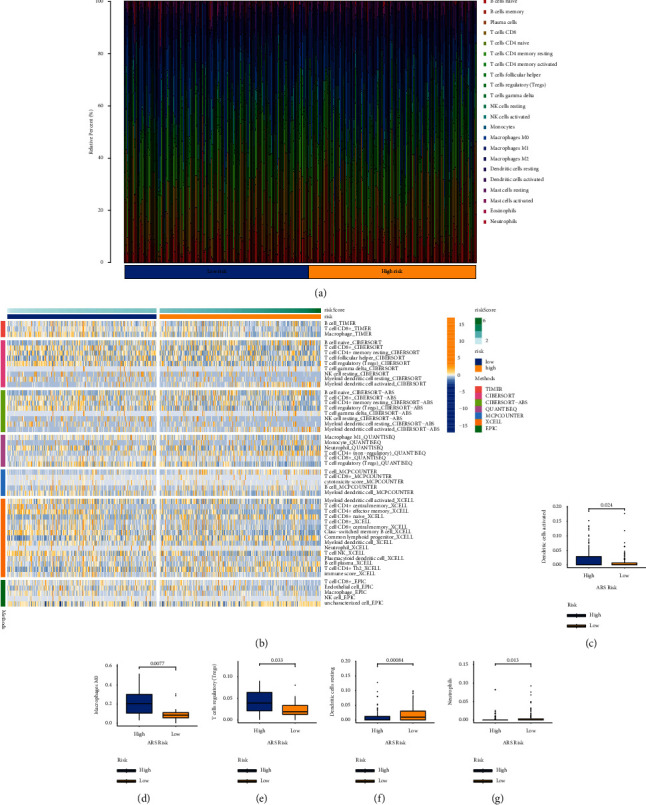
Immune landscape of the ARS. (a) Relative proportion of immune infiltration of the EC. (b) Heatmap for ARS and immune activity. (c–g) Box plots presenting remarkably immune cells between two groups.

To explore the correlations between ARS and immune cells infiltration, we carried out the CIBERSORT algorithm to analyze the proportion of the 22 immunocyte (Figure 6(a)). Afterward, the ssGSEA method was employed to mirror the status of immune microenvironment (TME) in EC (Figure 6(b)). We observed that activated dendritic cells, M0 macrophages, and T-cells regulatory (Tregs) were upregulated in the ARS-high group (Figures 6(a)–6(c)). However, ARS-low group had lower infiltration levels of neutrophils and resting dendritic cells (Figures 6(d) and 6(e)).

### 3.6. Correlation between ARS and Immune Checkpoint

**Figure 7:**
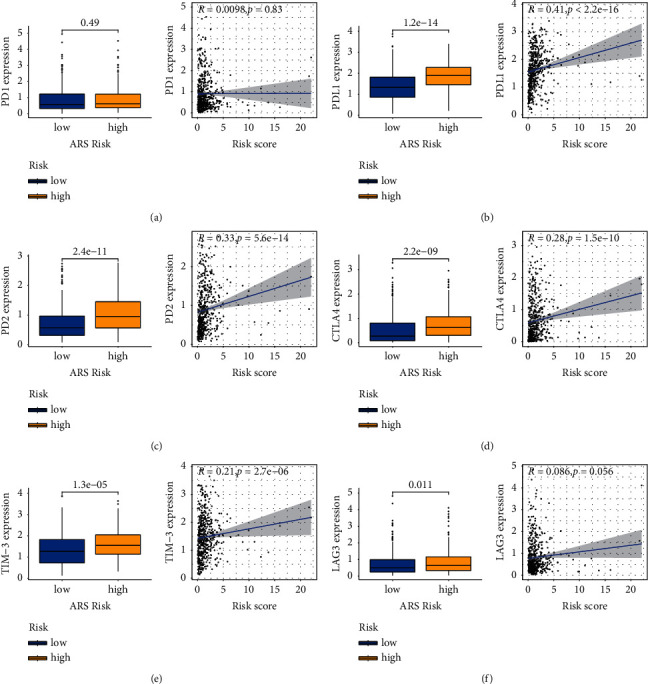
Analysis of immune checkpoints in EC. (a) PD1. (b) PD-L1. (c) PD-L2. (d) CTLA4. (e) TIM-3. (f) LAG3.

In view of the crucial role of checkpoint inhibitor-based immunotherapy, we analyzed the expression of immune checkpoint markers in the two groups. The results revealed that PD-L1, PD-L2, CTLA4, TIM-3, and LAG3 were positively correlated with ARS-risk (Figure 7). These results suggest that the ARS-high group is prone to generate an immunosuppressive microenvironment.

### 3.7. Analysis of Immunotherapy Response

**Figure 8:**
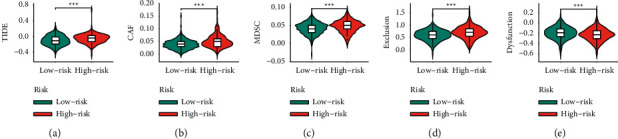
Analysis of immunotherapy response of EC patients. (a) TIDE score in two groups. (b) CAF score in two groups. (c) MDSC score in two groups. (d) T-cell exclusion score in two groups. (e) T-cell dysfunction score in two groups.

We further performed TIDE to estimate the efficacy of immunotherapy in two groups. Higher TIDE scores suggest a higher likelihood of immune evasion, indicating that patients may not benefit from immune checkpoint inhibitors (ICI) therapy. In our analyses, the ARS-high group displays a higher TIDE score than the ARS-low group (Figure 8(a)), pointing out that patients with high ARS score would not benefit from ICI therapy. Also, the ARS-high group had higher scores of CAF, MDSC, and exclusion, but the dysfunction score was lower in the ARS-high group (Figures 8(b)–8(e)).

### 3.8. Validation of the Hub Genes of the ARS Model

**Figure 9:**
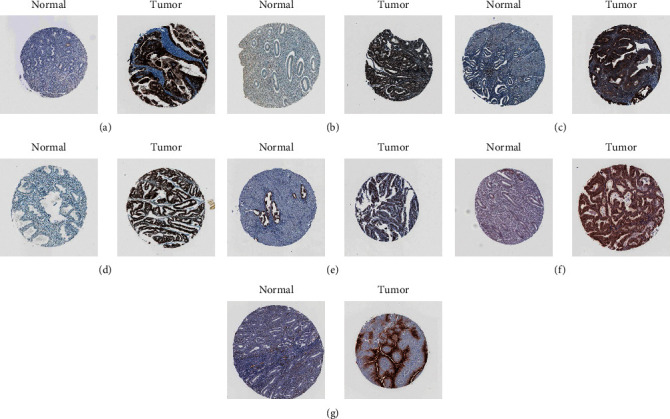
Validation of the hub genes of ARS. (a) CDKN2A. (b) E2F1. (c) ENDOG. (d) EZH2. (e) HMGA1. (f) PLK1. (g) SLC2A1.

Based on the HPA website, the expression patterns of model hub genes were confirmed by immunohistochemistry. The results are in line with our previous differential expression analysis. We found that all hub genes of ARS were highly expressed in EC cases (Figure 9).

## 4. Discussion

EC is one of the malignancies that imperils female's health, with a 5-year survival rate of 16% in patients with distant metastasis. This disease is hardly diagnosed at early stages due to a shortage of reliable prognosis-related biomarkers. Consequently, it is urgent to develop robust prognostic indicators to enhance the prediction of EC prognosis. Anoikis, a specific form of apoptotic cell death, was reported to regulate the biological behavior of various tumors. For example, CPT1A could confer anoikis resistance and facilitate metastasis in colorectal cancer (CRC) through the regulation of fatty acid oxidation [9]. Mo reported that IQGAP1 could enhance cell viability and inhibit anoikis by activating the Src/FAK pathway, indicating that it can be a reliable indicator for metastasis and prognosis of hepatocellular carcinoma (HCC) [12]. Moreover, CCN2 was proved to block lung cancer development via the DAPK-related anoikis pathway [13]. Thus, anoikis-related genes are prospective treatment targets and prognostic markers for tumors.

In this study, we first identified specific ARGs in EC based on the TCGA-UCEC project. To generate a robust ARGs-based signature for EC, all patients were separated into training and testing cohorts. After performing stepwise cox regression, we developed a seven-gene prognostic model in the training set. There is a marked difference in prognosis between two groups. And, the testing and the entire sets were used to confirm the accuracy of our proposed signature. To expand the performance of the ARS model, we generated an ARS-based nomogram that combined age, stage, grade, and risk score. The calibration plots show that the nomogram has a good fit for forecasting prognosis.

Our proposed ARS model was dramatically correlated with survival outcome of EC cases. The ARS consisted of seven anoikis-related genes, including CDKN2A, E2F1, ENDOG, EZH2, HMGA1, PLK1, and SLC2A1. All these signature genes have been proved to be closely bound up with tumors. For instance, Luo et al. found that CDKN2A was upregulated in hepatocellular carcinoma and associated with dismal prognosis [14]. As a transcription factor of the E2F family, E2F1 is shown to get involved in regulation of cell cycle. Xu et al. studied the expression of E2F1 in GC specimens and normal control and observed that overexpressed E2F1 could enhance TINCR expression at the transcription level, resulting in oncogenic growth in GC [15]. ENDOG, a nuclear-encoded endonuclease, affects cancer cell viability and tumor prognosis via the PI3K/PTEN axis. Notably, ENDOG silencing inhibited cell growth of endometrial cancer, thyroid carcinoma, and glioblastoma [16]. EZH2, a member of the polycomb-group (PcG) family, is reported to be a novel target for tumor control [17]. In ovarian cancer, EZH2 could retain cell stemness and confer chemoresistance by promoting CHK1 signaling [18]. Also, EZH2 mediates EMT of pancreatic cancer (PC) by binding with miR-139-5p [19]. Han et al. reported that HMGA1 was upregulated in EC cases and greatly associated with late stage. It could exert a carcinogenic effect in EC by targeting the Wnt/*β*-catenin pathway [20]. Gao showed that PLK1 affects cell proliferation and apoptosis by boosting MCM3 phosphorylation in renal cell carcinoma (RCC). SLC2A1, also named glucose transporter type 1 (GLUT1), could act as an oncogene to facilitate tumor development through the glycolysis pathway [21, 22].

To detect the potential biological pathways of the ARS, GSEA algorithm was applied. We found that various tumour signaling pathways were activated in the ARS-high group, such as IL2/STAT5 signaling, notch signaling, P53 signaling, PI3K/AKT/mTOR signaling, TGF *β* signaling, and Wnt/*β*-catenin signaling. All these pathways have been previously reported to get involved in EC growth and development. For instance, AP24534 might induce a significant reduction in STAT5 signaling on EC with higher FGFR2 mutations [23]. Guo et al. indicated that lncRNA-MEG3 has a lower expression in EC cases, and silencing MEG3 could restrict the cell viability by triggering the notch pathway [24]. It is well known that p53 could be used as a tumor suppressor gene inhibiting tumor initiation and development. Liu and his colleagues unearthed that downregulation of UBE2C could suppress estradiol-induced EMT by promoting p53 ubiquitination in EC, which provide a valuable therapeutic target for EC patients [25]. Likewise, the PI3K/AKT/mTOR pathway (PAM) also plays a crucial role in the tumorigenicity of EC. As revealed by Lin et al., silencing of FAM83 B could inhibit the activation of PAM, resulting in the suppression of EC growth and metastasis [26].

Recently, immunotherapy has been identified as a new treatment option for EC. The tumor microenvironment (TME), which consists of the ECM, stroma cells, tumour vasculature, and various cells of immune system, stimulates the initiation and progression of cancer [27, 28]. It is well known that immunosuppressive cells could induce the occurrence of immune escape in TME, which in turn facilitate tumor metastasis and progression. Tregs, a well-known type of immunosuppressive cells, has been proved to be correlated with prognosis of patients, indicating that Treg count might be an effective prognostic marker for EC [29]. In addition, Li and his colleagues reported that Treg has a higher infiltration level in in the peripheral blood of EC cases than healthy control [30]. Also, cancer-associated fibroblast (CAF) is a major cell subpopulation in TME which affects the biological behavior of EC through CAF-tumor cell cross-talk [31]. Furthermore, CAF extracted from EC tissues could secrete IL-6, which subsequently boosts c-Myc expression to accelerate EC growth [32]. In our study, the ARS-high group has higher infiltration levels of Tregs and CAFs, pointing out that patients with high ARS score are more likely in state of cancer immunosuppression.

We further detected whether ARS could offer valuable reference for immunotherapy response. Immune checkpoints are the classical molecules used to assess the efficacy of immunotherapy. Our analyses show that the PD-L1, PD-L2, CTLA4, TIM-3, and LAG3 were greatly enriched in the ARS-high group. Moreover, we found that the ARS-low group has a lower TIDE score. All these results pointed out that patients with high ARS score would not benefit from ICI therapy.

Although our proposed ARS displays a powerful performance in forecasting prognosis of EC patients, the present subject has some limitations. Firstly, all clinical cohorts of EC cases analyzed in the current study were exclusively obtained from the TCGA website. The potency of the ARS model also needs to be verified by external cohorts. Second, we will further confirm the expression pattern of ARS genes by our local clinical specimens by immunohistochemistry. Moreover, the underlying mechanisms of ARS genes should be explored based on experimental analyses.

In summary, our study developed a novel anoikis-related signature in EC. The ARS of both biomarkers could ameliorate the prediction of EC survival outcome and reflect the immune conditions and estimate the immunotherapy response for EC patients. Our study brings a novel perspective to the therapeutic strategy for patients with EC.

## Figures and Tables

**Table 1:** Clinicopathologic characteristics of EC patients.

Features	Training set	Validation set	Entire set
Total	260 (100%)	260 (100%)	520 (100%)

*Age*			
≤60	99 (37.79%)	102 (39.23%)	201 (38.51%)
>65	163 (62.21%)	158 (60.77%)	321 (61.49%)

*Stage*			
I-II	185 (70.61%)	192 (73.85%)	377 (72.22%)
III-IV	77 (29.39%)	68 (26.15%)	145 (27.78%)

*Grade*			
G1-G2	118 (45.04%)	94 (36.15%)	212 (40.61%)
G3-G4	144 (54.96%)	166 (63.85%)	310 (59.39%)

**Table 2:** Seven anoikis-related signatures greatly correlated with survival outcome.

Gene	Coefficient	Hazard ratio (95% CI)	*P* value
CDKN2A	0.1068	1.29 (1.16–1.45)	<0.001
E2F1	0.0941	1.48 (1.21–1.82)	<0.001
ENDOG	-0.2316	0.71 (0.54–0.92)	0.011
EZH2	0.1626	1.52 (1.12–2.07)	0.007
HMGA1	0.8872	1.26 (1.04–1.53)	0.012
PLK1	0.1673	1.36 (1.07–1.73)	0.011
SLC2A1	0.0228	1.22 (1.02–1.46)	0.028

## Data Availability

The public datasets to support the results of this subject can be gained from TCGA (https://portal.gdc.cancer.gov/), HPA (https://www.proteinatlas.org/), and TIDE (https://www.proteinatlas.org/).

## Supplementary Materials

Supplementary Materials
*Supplementary Table 1*. A list of 434 anoikis-related genes (ARGs).
