# Supplementary material for: Development of Biomarker Signatures Associated with Anoikis to Predict Prognosis in Endometrial Carcinoma Patients
Source: J Oncol. 2021 Dec 28;2021:3375297. doi: 10.1155/2021/3375297 (PMC8727165; doi:10.1155/2021/3375297)
Supplement: Supplementary Materials — Supplementary Table 1. A list of 434 anoikis-related genes (ARGs). [file 3375297.f1.pdf]

Supplementary Table 1: The list of 434 anoikis-related genes (ARGs).

Gene Symbol	Relevance score
BRMS1	14.54911
PTK2	7.193454
NTRK2	7.183176
BCL2L11	6.624886
SRC	6.104873
CEACAM6	6.054066
CAV1	5.393483
AKT1	5.331155
ITGB1	4.938623
CEACAM5	4.617144
BCL2	4.483292
CASP8	4.430302
EGFR	4.384342
PTRH2	4.139895
STAT3	4.086776
SIK1	4.038517
DAPK2	3.958509
CTNNB1	3.94576
ZNF304	3.910815
MAPK1	3.685743
BMF	3.685458
ITGA5	3.630884
MCL1	3.533736
TP53	3.451738
BCL2L1	3.319262
CASP3	3.079218
CDH1	3.019521
BAD	2.92408
PIK3CA	2.905329
PAK1	2.892794
ITGAV	2.837788
MAPK3	2.694114
PTGS2	2.656932
BAX	2.514714
FN1	2.508724
BCAR1	2.503318
PTEN	2.48593
TLE1	2.447864
PDK4	2.405831
ERBB2	2.401457
ANGPTL4	2.367288

CYCS	2.308967
BRAF	2.304839
ANXA5	2.236495
BIRC5	2.226358
MTOR	2.221419
TIMP1	2.215896
ITGA2	2.192107
BDNF	2.178729
CSPG4	2.166408
BSG	2.166408
AKT2	2.154229
STK11	2.12343
IGF1	2.119635
IGF1R	2.118469
ITGA6	2.082232
ILK	2.055982
CFLAR	2.055758
RHOA	2.041556
HIF1A	2.037955
DAP3	2.028509
MYBBP1A	1.995728
TLE5	1.97341
ITGA3	1.971719
PTK2B	1.969084
CCND1	1.955544
CTTN	1.955544
CALR	1.919438
CDCP1	1.906433
CHEK2	1.883697
SKP2	1.883073
HGF	1.858571
E2F1	1.856835
EGF	1.846404
PIK3CG	1.843222
YAP1	1.842585
ITGB4	1.831272
DAPK1	1.821627
PIK3R1	1.797109
MAP2K1	1.77256
CXCL12	1.753584
LGALS3	1.71865
BAK1	1.706999
ABHD4	1.684149

CD44	1.680662
ITGA4	1.66762
FADD	1.66762
TGFB1	1.66342
HMCN1	1.66342
MMP2	1.650489
CDKN3	1.645226
CASP9	1.632686
MTDH	1.632686
TNFRSF10B	1.614124
CXCL8	1.614124
MIR200C	1.614124
AR	1.59767
CDKN2A	1.594698
MAPK8	1.594698
PIK3CB	1.594698
CLDN1	1.594698
MIR26A1	1.594698
MIR204	1.594698
CDKN1B	1.574273
ATF4	1.574273
KLF12	1.574273
MYC	1.552678
PLAU	1.552678
PLK1	1.552678
SMAD4	1.552678
CDKN1A	1.552678
MUC1	1.552678
PLAUR	1.552678
LGALS1	1.552678
PYCARD	1.552678
SESN2	1.552678
NTRK1	1.552652
KRAS	1.54763
ITGB3	1.54763
BID	1.529686
THBS1	1.529686
HRAS	1.514663
CDK11B	1.504984
CDK11A	1.504984
XIAP	1.497984
PPARG	1.478122
IL6	1.478122

MIR145	1.478122
CCR7	1.44841
MSLN	1.44841
RAC1	1.442192
GRHL2	1.442192
NOTCH1	1.42001
RHOG	1.417655
CCAR2	1.417655
NQO1	1.414685
BIRC3	1.412019
MMP13	1.380174
FAS	1.377181
MTA1	1.377181
MYO5A	1.374681
EDA2R	1.374681
CCN6	1.374681
ABL1	1.356228
MMP9	1.356228
MAPK11	1.356228
PTHLH	1.352479
GLI2	1.335803
CXCR4	1.325617
RIPK1	1.317119
HMGA1	1.314208
TNFSF10	1.314208
SIK2	1.314208
ETV4	1.291216
S100A4	1.291216
NTF3	1.291216
MIR21	1.291216
MIR124-1	1.291216
LATS1	1.266514
HTRA1	1.266514
CEACAM3	1.266514
EIF2AK3	1.263623
LAMB3	1.263623
LAMC2	1.263623
LAMA3	1.263623
CDH2	1.244197
CSNK2A1	1.244197
EDIL3	1.244197
EZH2	1.242513
ZEB2	1.239652

TLN1	1.239652
EPHA2	1.223772
SOD2	1.223772
SIRT3	1.223772
OLFM3	1.223772
CEMIP	1.223772
FBXW7-AS1	1.222176
CLU	1.20994
SPINK1	1.20994
CPEB2	1.20994
TSG101	1.202178
MIR200A	1.202178
SERPINA1	1.196685
AKT3	1.179185
RELA	1.179185
PRKCA	1.179185
TNFRSF1A	1.179185
FASLG	1.179185
AFP	1.179185
CEBPB	1.179185
SATB1	1.179185
EEF1A1	1.179185
ITGA8	1.179185
PBK	1.179185
LTB4R2	1.179185
CD63	1.179185
NOX4	1.179185
MAVS	1.179185
RHOB	1.176215
CCN2	1.176215
PPP1R13B	1.176215
PLG	1.170042
RAF1	1.154483
PRKCQ	1.154483
BRCA2	1.154483
PARP1	1.154483
DOCK1	1.154483
SP1	1.154483
HAVCR2	1.154483
VTN	1.154483
INHBB	1.154483
RANBP9	1.154483
PDCD4	1.154483

PRPF4B	1.154483
SESN1	1.154483
SESN3	1.154483
PHLDA2	1.154483
ZBTB7A	1.154483
CD24	1.154483
MIR141	1.154483
MET	1.153404
ELANE	1.136212
KDR	1.127622
MDM2	1.127622
PRKCI	1.127622
NFE2L2	1.127622
RB1	1.127622
FGF2	1.127622
HK2	1.127622
KL	1.127622
CRYAB	1.127622
EPHB6	1.127622
IQGAP1	1.127622
LTF	1.127622
SDCBP	1.127622
SPIB	1.127622
MGAT5	1.127622
ABHD2	1.127622
TRIM31	1.127622
MIR1827	1.127622
PDGFRB	1.097909
TLR3	1.097909
ROCK1	1.097909
CPT1A	1.097909
NRAS	1.097909
PLAT	1.097909
CASP10	1.097909
PAK4	1.097909
VEGFA	1.097909
ZEB1	1.097909
PIN1	1.097909
UBE2C	1.097909
YWHAZ	1.097909
TWIST1	1.097909
ELK1	1.097909
BMP6	1.097909

PRDX4	1.097909
BNIP3	1.097909
BNIP3L	1.097909
KDM3A	1.097909
LMO3	1.097909
ZNF32	1.097909
MIR200B	1.097909
MIR363	1.097909
MIR525	1.097909
TUBB3	1.084078
PTPN11	1.064185
SLC2A1	1.064185
HMOX1	1.064185
PRKACA	1.064185
CBL	1.064185
PAK3	1.064185
PIK3R2	1.064185
CASP6	1.064185
PPP2CA	1.064185
CD36	1.064185
CDH3	1.064185
LRP1	1.064185
PTK6	1.064185
EEF2K	1.064185
GLO1	1.064185
PAK2	1.064185
LPAR1	1.064185
SFN	1.064185
ADCY10	1.064185
TRAF2	1.064185
PIK3R3	1.064185
RBL2	1.064185
SIRPA	1.064185
TNFRSF12A	1.064185
CEACAM1	1.064185
BAG1	1.064185
APOBEC3G	1.064185
GDF2	1.064185
MNX1	1.064185
VPS37A	1.064185
COL13A1	1.064185
RAD9A	1.064185
IFI27	1.064185

ITPRIP	1.064185
BCL2L15	1.064185
SNAI2	1.037513
GLUD1	1.024181
MYH9	1.024181
NOTCH3	1.024181
PTPN1	1.024181
TPM1	1.024181
FASN	1.024181
RPS6KB1	1.024181
SIRT1	1.024181
PPP2R1A	1.024181
CD151	1.024181
CTNND1	1.024181
MMP11	1.024181
COL4A2	1.024181
ARHGEF7	1.024181
PPP2R5A	1.024181
BST2	1.024181
PPP2R2D	1.024181
CCN1	1.024181
CCDC178	1.024181
MIR30C1	1.024181
MIR30B	1.024181
MIR10A	1.024181
SHC1	1.002452
BUB1	0.972047
CDC25C	0.972047
BUB3	0.972047
FER	0.972047
SETD2	0.972047
CDK1	0.972047
ITGB5	0.972047
TP73	0.972047
MAD2L1	0.972047
BCL2L2	0.972047
SLCO1B3	0.972047
DLG1	0.972047
PDCD6IP	0.972047
SCRIB	0.972047
TDGF1	0.972047
EDAR	0.972047
SH3GLB1	0.972047

DYNLL2	0.972047
TSC2	0.947214
BAG4	0.908719
MAP3K7	0.90721
F10	0.846184
HSP90B1	0.846184
F3	0.846184
ADAMTSL1	0.846184
SERPINB1	0.846184
MIR181A1	0.846184
MAP3K1	0.827443
CTBP1	0.827443
CEACAM4	0.793718
PXN	0.78051
MALAT1	0.776761
IKBKG	0.729213
TFDP1	0.729213
CRYBA1	0.729213
ANKRD13C	0.729213
SERPINE1	0.720187
FOXO3	0.71893
ACTG1	0.70158
ARHGDIA	0.70158
EZR	0.70158
SLC39A6	0.70158
BIN1	0.690797
TIAM1	0.690797
PDPK1	0.686462
SMAD7	0.663935
RHOC	0.634223
CASP2	0.62646
TNC	0.603468
IRF6	0.603468
NTRK3	0.600498
GNE	0.578766
XAF1	0.578766
SFRP1	0.575717
MAP2K2	0.560494
CSK	0.560494
PIK3C2B	0.560494
FOXC2	0.560494
ARHGDIB	0.560494
ENDOG	0.560494

TAGLN	0.560494
FBLIM1	0.560494
RACK1	0.560494
CCDC80	0.560494
ANGPTL2	0.560494
HOTAIR	0.560494
PRKD1	0.551904
LDHA	0.522192
ANXA2	0.522192
SPP1	0.522192
SMARCE1	0.522192
RBFOX2	0.522192
QSOX1	0.522192
RPS6KA3	0.488467
CDC42	0.488467
MAOA	0.488467
PIP5K1C	0.488467
JUP	0.488467
ATF2	0.488467
NKX2-1	0.488467
OCLN	0.488467
ID2	0.488467
CRABP2	0.488467
CEACAM8	0.488467
PITPNC1	0.488467
AFAP1L1	0.488467
HSPB1	0.448463
PCNA	0.448463
GSK3B	0.448463
NGF	0.448463
TP63	0.448463
CTNNA1	0.448463
KRT14	0.448463
SPHK1	0.448463
EHMT2	0.448463
RAC3	0.448463
SIRT6	0.448463
OGT	0.448463
NDRG1	0.448463
STK38	0.448463
ACP1	0.448463
FOXA1	0.448463
RHOQ	0.448463

ONECUT1	0.448463
S100A7	0.448463
SRSF3	0.448463
GKN1	0.448463
MIR107	0.448463
MIR630	0.448463
